# The Employment of Digitaline in Spormatorrhœa

**Published:** 1853-06

**Authors:** M. Lucien Corvisart


Translated from the French for the N. W. Med. and Surg. Journal.

ART. V.—*The Employment of Digitaline in Spormatorrhœa.* By M. LUCIEN CORVISART.

M. L. CORVISART, having had the boldness to introduce the use of digitaline in spormatorrhœa, has already used it three times and each time with success. Without pretending to claim from the observations already made, the conviction of practitioners, he thinks, and justly, as it seems to us, that this medicine deserves attention.

We have here the first case in which he thought he perceived benefit from the use of digitaline in seminal flux.

CASE I.—P. M., aged 20 years, a stamper, entered the Hotel-Dieu the 1st of Feb. 1848, in the ward Saint Agnes, under the care of Professor Chomel—Appearance feeble, pale, observing during the week strict continence, but every Sunday for more than a year entering into the lowest debauchery and repeating coition five or six times, digestion bad, anorexia, palpitation of the heart, face bloated and hot, dizziness, tingling of the ears, accasional attacks of dyspnœa compelling the patient to walk his room during the night, disturbed dreams, and strange noises during partial sleep. The above symptoms had supervened since November last, at which time, after a debauch, he experienced a severe pain in the precordial region, characteristic of endo-cardites, for which he was subjected at the Hotel-Dieu to a strictly antiphlogistic treatment, (three bleedings, &c.) The patient, being unable to continue his labors, has now returned. The volume of the heart is normal; the blow of the point against the walls of the chest with a *bruit de souffle*, somewhat dry at the first sound of the heart. M. Chomel prescribed first 1, then 2 milligrammes of digitaline.

The sixth day of the treatment, the palpitations having very much diminished, the patient mentioned that since November he had almost every night involuntary pollutions, often leaving no trace in his memory—they were known only by the stains on his linen. When awakened by the pollutions, the penis was found to be in erection, as was also the case each morning.

Pollutions take place almost every day, under the influence of erotic ideas, or the simple friction of the clothes, with erection and ejaculation, but without lively pleasure; they sometimes occur during defecation.

The patient, for three months, has not seen a woman.

He is astonished that the emissions have not occurred for the last three days, (now the ninth since entering the hospital, and the third of the treatment of the palpitations by digitaline.)

Feb. 13th, one pollution without any remembrance of it; the 14th one on waking; the 15th one on waking, very slight, (one or two drops); 3 milligrammes were ordered; no spormatorrhœa from the 16 to to the 19th; then seven days entire without a single pollution, although each day the patient examined his shirt and drawers with the greatest care; this was also done several times by myself.

March 7th—One emission on waking.

March 12th—Discharged, no pollution since the last date.

After thirty eight days the patient had had but five pollutions, and for twenty two days but one; he was still troubled with erections, although less than formerly. The palpitations were very much diminished.

CASE II.--L. H., aged 18 years, sculptor, having never indulged in venereal excesses, was taken when between fifteen and sixteen years with nocturnal emission without pleasurable sensations. At first these accidents occurred almost every night from his sixteenth to his seventeenth year.

The young patient, very much affected, bound the penis with pack thread, bathed the parts in ice-cold water, avoided every occasion of provoking erotic ideas, and lived in the most absolute continence.

About the month of December, 1847, he commenced the practise of masturbation two or three times a week. During the two months in which he indulged in this habit, the nocturnal pollutions disappeared almost entirely; nevertheless the symptoms, which had already begun to manifest themselves, increased. The strength was diminished, the spirits depressed, the memory short, the patient became very sensible to cold, digestion slow and painful, the appetite greedy. About the end of Feb. 1848, the patient abandoned his practices and renounced all venereal pleasures. A few days after the nocturnal emissions returned as frequently as before. The erections when the patient was awakened, (which was rarely the case,) were but slight; they were not always accompanied by erotic ideas, but sometimes even the reverse.

L. H. remarked that for some weeks, about the close of micturition, the urine was loaded with a white albuminous cloud, which sunk to the bottom of the vessel, and was more abundant in the morning after fatigue.

The patient experienced pain along the spinal column, shooting into the epigastric region and a general, although light, habitual trembling with stammering.

The patient had used, without success, camphor, internally and externally; he thought the erections more numerous, and the emissions more frequent. M. Huguier had prescribed, about the end of March, bitter drinks, the syrup of gentian, exercise, &c., The pollutions diminished during one or two weeks and then returned,

On the 13th of April the patient came to consult M. Huguier, who at my request had the goodness to prescribe the digitaline, 3 milligr. a day. Each of the three following days the pollutions persisted, then an interval of six days without any. The 23d of April he returned to know whether he should continue the use of the digitaline. M. Huguier admitted him to his wards in the hospital Beaujon, in order the better to observe the case. The seventeenth day of the treatment, which was continued, he had one pollution, that was all. The patient was discharged the 15th of May, having had in thirty-four days but five pollutions, of which the two last were separated by an interval of twenty-two days. There was no seminal discharge in the urine, that fluid being scarcely troubled and containing no spermatic animalcula. He had neither trembling nor stammering; he was free from pain along the spine; his strength improving, and his digestion good.

The third case is too lengthy to present entire, but it is not less conclusive than the others.

How shall the action of digitaline be explained? The first patient appears to be an example of spermatorrhœa from atony of the genital organs, this was the case also in the third, but certainly not in the second, who appears to have possessed remarkable temperance. In two of the cases the erections were light, not insupportable. It is not by calming these, then, that the remedy acts. So far, its administration is purely empirical, but this is not sufficient reason for disregarding it.

